# Genome-wide analysis of MADS-box families and their expressions in flower organs development of pineapple (*Ananas comosus* (L.) Merr.)

**DOI:** 10.3389/fpls.2022.948587

**Published:** 2022-10-12

**Authors:** Xiaolu Pan, Yanwei Ouyang, Yongzan Wei, Bencheng Zhang, Jing Wang, Hongna Zhang

**Affiliations:** Key Laboratory for Quality Regulation of Tropical Horticultural Crops of Hainan Province, College of Horticulture, Sanya Nanfan Research Institute, Hainan University, Haikou, China

**Keywords:** pineapple (*Ananas comosus* (L.) Merr.), MADS-box, expression profiling, floral organ, syntenic analysis

## Abstract

MADS-box genes play crucial roles in plant vegetative and reproductive growth, better development of inflorescences, flower, and fruit. Pineapple is a typical collective fruit, and a comprehensive analysis of the MADS-box gene family in the development of floral organs of pineapple is still lacking. In this study, the whole-genome survey and expression profiling of the MADS-box family in pineapple were introduced. Forty-four *AcMADS* genes were identified in pineapple, 39 of them were located on 18 chromosomes and five genes were distributed in five scaffolds. Twenty-two *AcMADS* genes were defined as 15 pairs of segmental duplication events. Most members of the type II subfamily of *AcMADS* genes had higher expression levels in floral organs compared with type I subfamily, thereby suggesting that *AcMADS* of type II may play more crucial roles in the development of floral organs of pineapple. Six *AcMADS* genes have significant tissue-specificity expression, thereby suggesting that they may participate in the formation of one or more floral organs. This study provides valuable insights into the role of MADS-box gene family in the floral organ development of pineapple.

## Introduction

The MADS-box transcription factors are one of the important transcription factor families in higher plants that play fundamental roles during plant development and floral organ differentiation (Alvarez-Buylla et al., 2000; Saedler et al., 2001). The prominent feature of the MADS-domain proteins is its MADS domain that consists of 56–58 amino acids (Shore and Sharrocks, 1995). The MADS domain can recognize the CArG-box with similar 10-bp A/T-rich DNA sequences (Folter and Angenent, 2006). In plants, MADS-box genes can be classified into two distinct groups, namely, types I and II, on the basis of the evolutionary relationships: type I members are SRF-like in plant, which only have a MADS (M) domain; type II MADS-box proteins are MEF2-like in plant, animal, and yeast, which contain a highly conservative DNA-binding domain (M), an intervening (I) domain, a semi-conservative K domain, and a C-terminal region (De et al., 2003; Smaczniak et al., 2012). The type I proteins are further divided into Mα, Mβ, and Mγ subfamilies, and the type II group also are defined as MIKC-type proteins, which comprise MIKC^C^-type and MIKC*-type proteins (Yang and Jack, 2004; Nam et al., 2004).

Flowering is a complex process that requires the cooperation and interaction of numerous genes. Previous reports have shown that the MADS-box genes can regulate the characteristics of floral meristems (Riechmann and Meyerowitz, 1997; Jongmin et al., 2003). Flowers are frequently arranged by four different kinds of organs arranged in whorls, with sepals in the first round, petals in the second whorl, stamens in the third round and carpels in the fourth whorl (Günter et al., 2016). The ABCDE model completely explains the individual development of plant flowers and the determination of the identity of floral organs (Theissen et al., 2000; Ma, 2000). Class-A genes determine the sepal development, petal development requires Class-A and Class-B genes, stamen development needs Class-B and Class-C genes to work together, carpel development is ascertained by Class-C genes, and ovule development is identified by Class-C and Class-D genes (Ma, 2000). Class-E genes need to assist other genes to participate in the determination of all flower organs and meristems (Hugouvieux et al., 2018). In *Arabidopsis*, almost every gene from this model, such as A (*APETALA1*) (Wang et al., 2014), B (*PISTILLATA*, *AP3*) (Jack et al., 1992), C (*AGAMOUS*) (Hugouvieux et al., 2018), D (*AGAMOUS-LIKE 11*) (Pinyopich et al., 2003), and E (*SEPALLATA 1, 2, 3, 4*) (Ditta et al., 2004), belong to the type II MADS-box subfamily. In addition, one of the main characteristics of MADS-box transcription factors is their distinct tetrameric protein complexes composed of MIKC-type MADS domain proteins, which control gene expression and hence floral organs identity during development (Theißen, 2001). MADS protein bind as dimers to DNA sequences called ‘CArG-boxes’ (Theißen and Gramzow, 2016). According to the FQM, the two protein dimers of each tetramer recognize two different CArG-boxes and bring them close to each other by DNA cyclization between the CArG-boxes (Theißen, 2001; Theißen and Saedler, 2001). In recent years, type II proteins have been increasingly recognized for their remarkable ability to form multimeric transcription factor complexes and their importance in plant development and evolution. Thereby suggesting that type II MADS-box genes play a vital role in the control of floral organ development. Pineapple is one of important tropical fruits with great economic and research value (Bartholomew et al., 2002; Ming et al., 2015; Lobo and Paull, 2017). This fruit is a typical collective fruit, and each floret forms a separate little fruitlet, which is gathered on the enlarged torus. When the plant height surpasses 35 cm and the number of leaves exceeds 35, the certain concentrations of ethephon are used to induce pineapple to bloom. The spike of the pineapple is generally composed of 50 to 200 flowerlets. The first flower blossoms on the bottom of the spike, followed by other flowerlets unfold from the bottom to the top. The flower is hermaphroditic and consists of one pistil, six stamens, three petals and three calyxes. The petal is about 2 cm in length, with lavender-coloured upper part and white lower part. The entire florescence lasts for 10–15 days, during which all petals fall or become dried and the fruit starts to develop (Zhang et al., 2016).

At present, few studies have been conducted on the morphological and physiological basis of collective fruits, especially the molecular mechanism. The physiological basis and molecular mechanism of the formation of collective fruits in pineapple should be understood. Previous studies have shown that MADS-box genes are involved in various physiological processes, especially with the identification of floral organs. Thus, the exploration of the function of MADS-box genes and the molecular mechanism of pineapple flower organ development has received considerable interest. In the present study, a comprehensive analysis, including the chromosomal localization, synteny analysis, and gene duplication, was conducted on the basis of the pineapple genome (Ming et al., 2015). Global expression analysis of MADS-box genes in different tissues and floral organs has been conducted by using RNA sequencing (RNA-Seq) and quantitative real-time polymerase chain reaction (qRT-PCR) to identify the specific MADS-box genes involved in the different biological processes. Six AcMADS genes demonstrate significant tissue specificity in different floral organs. These initiatives provide a reference for the functions of MADS-box genes in pineapple.

## Materials and methods

### Plant materials and treatments

The pineapple plants (*Ananas comosus* L. cv. Comte de Paris) used in this study were grown in South Subtropical Crop Research Institute, Zhanjiang, China (21°10′2″N; 110°16′34″E). About 200 homogenous plants (20-month-old) were induced by ethylene in early October 2019. The experimental groups were treated with 30 ml of 400 mg·L^− 1^ ethylene perfusion in the center of the pineapple, the control group used 30 ml water instead of ethylene. The terminal buds, roots and leaf of pineapples plants were respectively collected before treatment. The entirely inflorescence at early spike stage (42 days after treatment) was collected and used to analyze gene expression characteristics in the different tissues of pineapple. And the floral organs of the pineapple flower, including petals, ovaries, stamens, sepals, and styles were also collected from twenty flowerlets in the middle of spike at 56 days after treatment (full-bloom stage). All samples were immediately frozen in liquid nitrogen and stored at −80°C until further use. All samples were performed with three biological replications. All treated tissue samples were frozen in liquid nitrogen as quickly as possible and stored at −80°C.

### Total RNA isolation and qRT-PCR

The total RNA was extracted from the pineapple tissues with the Huayueyang RNA extraction kit (Huayueyang, China) according to the manufacturer’s instructions. The concentration and quality of all purified RNA were checked on 1% agarose gel and Bio Photometer Plus (Eppendorf, Germany). RNA (5 μg) was reverse transcribed to cDNA with the Revert Aid First-Strand cDNA Synthesis Kit (Thermo Fisher Scientific, USA).

The quantitative RT-PCR assays were conducted in the Light Cycler 480 II (Roche, Switzerland) by using SYBR Green qPCR Master Mixes (Thermo Fisher Scientific, USA). *AcActin* gene was used as the internal control of pineapple (Azam et al., 2018). The reaction mixture included 5 μL of 2× SYBR Green PCR Master Mix (Applied Biosystems), a diluted cDNA template of 1 μL, and 1 μL of each primer in a final volume of 10 μL. The qPCR conditions were as follows: 50°C for 2 min, 95°C for 2 min, 45 cycles of 15 s at 95°C, 56°C for 15 s, and 72°C for 40 s. The 2^−ΔΔCt^ (Livak and Schmittgen, 2001) method was used to calculate the relative expression levels of each gene. All quantitative PCRs were performed with three biological replications. All primers were designed by the Primer Premier 5.0 (Lalitha, 2000) and listed in Additional file 4.

### Database search and MADS-box gene family identification in pineapple

The nucleotide and protein sequences of AtMADS genes were searched and obtained from TAIR (http://www.arabidopsis.org/) databases. This research investigated the MADS proteins of pineapple, *Arabidopsis*, grape, banana, rice, and maize plants. We downloaded the HMM file keeping with the MADS domain (PF00319) from the Pfam protein database (http://pfam.xfam.org, Pfam 31.0) and searched for the MADS-box genes in the pineapple genome database through HMMER 3.0. The e-values lower than 0.01 and the default parameters were selected. The MADS-box core sequences were confirmed by using the SMART database and the NCBI CDD web server (http://www.ncbi.nlm.nih.gov/Structure/cdd/wrpsb.cgi). Sequences without MADS-box domain were deleted. The length of sequences, molecular weight, and isoelectric point (PI) of the MADS proteins were obtained by using the compute pI/Mw tool in the ExPASy server (http://web.expasy.org/protparam/). The subcellular localization of the identified MADS proteins was predicted by the cello web server (http://cello.life.nctu.edu.tw/).

### Phylogenetic tree construction and classification in pineapple MADS-box genes

MADS-box genes of *Arabidopsis* and grape were used as a reference to classify the MADS-box genes of pineapple. A single alignment of pineapple MADS domain by using the Clustal W program was built in MEGA6.0 software; a phylogenetic tree was then constructed by using maximum likelihood (ML) method (Tamura et al., 2013) with the following parameters: 1000 bootstrap replications, partial deletion, and Jones–Taylor–Thornton (JTT) + gamma distributed (G) model.

#### Chromosomal location, gene duplication, and syntenic analysis

The physical positions of the *AcMADS* genes on chromosomes were identified with TBtools (Chen et al., 2020) according to the gene location in the pineapple genome. The tandem duplication events were defined as the single chromosomal region contiguous homologous genes with the original repeat, while the duplicate of the whole blocks of genes between different chromosomes was defined as segmental duplication (Leister, 2014). Gene duplication events were drafted with Multiple Collinearity Scan tool kit (MCScanX) (Wang et al., 2012). In the syntenic analysis, the genome data of five representative species were downloaded from Ensemble plants database, and the diagrams were visualized using the TBtools with Dual Synteny Plotter (Chen et al., 2020).

### *cis*-element analysis of *AcMADS* promoter sequences

A 2000-bp upstream sequence from the translation initiation codon of each *AcMADS* gene was gained in genomic data of pineapple (http://pineapple.angiosperms. org/pineapple/html/index.html) to explore the function of *AcMADS* genes. All *cis*-element were calculated on the regions of promoter by the PlantCARE online tool (http://bioinformatics.psb.ugent.be/webtools/plantcare/html/) (Lescot et al., 2002).

### Expression profiling of *AcMADS* genes by RNA-seq

To found the expression profile of *AcMADS* genes linked with floral organ development of pineapple. The aforementioned 30 tissue samples were transported to Gene Denovo Company (Guangzhou, China) and sequencing was performed on Illumina Hiseq platform. The transcript abundance of each gene was computed by FPKM values (fragments per kilobase of repeat per million fragments mapped) (Trapnell et al., 2010). Genes with log2 and p-value were hierarchical clustered. Heatmaps of the RNA-seq data were generated using TBtools software (Chen et al., 2020). And the transcriptome data have uploaded into National Genomics Data Center database (https://ngdc.cncb.ac.cn/). The assigned accession of the submission is: CRA006826.

### The protein-protein interaction networks functional enrichment analysis of AcMADS

In order to further explore the function of key AcMADS genes in flower organ development of pineapple, the protein sequence of AcAGL11c, AcANR1b, AcAGL11a, AcBS, AcFLC2 and AcAGL11b were uploaded to the STRING database (https://cn.string-db.org/), the interaction network which these putative AcMADS genes involved in were investigated based on the orthogous genes between pineapple and *Arabidopsis.*

## Results

### Characteristics of pineapple flower

**Figure 1:**
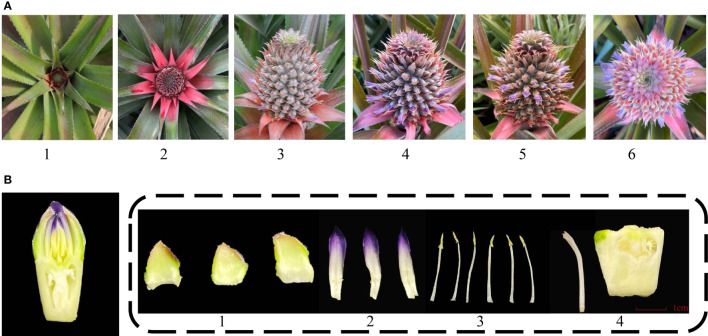
Different stages and organs of pineapple floral development. **(A)** Different developmental stages of inflorescence. 1. Inflorescence emerging; 2. Inflorescence swelling out; 3. Early flowering stage; 4. Full-bloom stage; 5. Flower fading stage; 6. Panoramic view of the full-bloom stage. **(B)** Floral organ structure of pineapple. 1. Sepal; 2. Petal; 3. Stamen; 4. Pistil.

The inflorescence of pineapple emerges from leaf clumps and looks like a pinecone. The entire inflorescence development to bloom is approximately 1 month. The spike of pineapple is composed of 50 to 200 florets. The blossoming order of the spike is from the bottom to the top (**Figure 1A**) (Zhang et al., 2016). The pineapple flowers are hermaphrodite and consist of three sepals, three petals, five stamens, and one pistil (**Figure 1B**). The edible part of pineapple involves the fleshy axis of inflorescence and the ovary of florets.

### Identification and classification of MADS-box genes in pineapple

**Figure 2:**
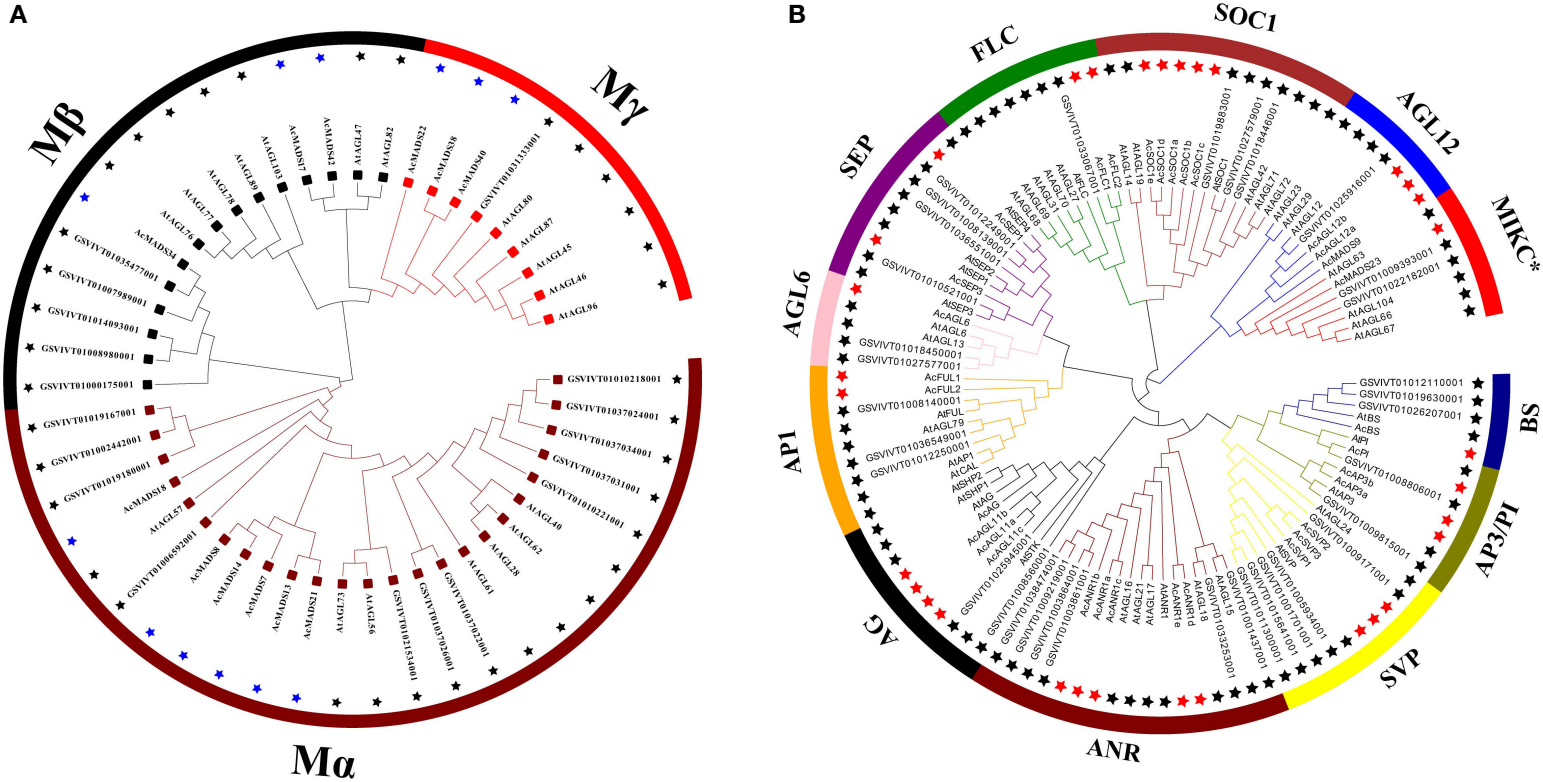
Phylogenetic analysis of type I **(A)** and type II **(B)** MADS-box genes in *Arabidopsis*, grape, and pineapple. The phylogenetic trees were constructed using the ML method. The black stars represent the MADS-box proteins from the grape and *Arabidopsis*, then the blue and red stars respectively represent the type I and type II MADS-box proteins from the pineapple. MADS-box proteins from the grape with the prefix “GSVIVT” indicate “VvMADS” and “At” means “AtMADS” in *Arabidopsis*.

The MADS-box protein sequences were used to Hidden Markov Model (HMM) search, and 54 candidate genes were originally obtained. Ten erroneously predicted MADS-box genes were removed. Finally, 44 MADS-box genes were selected and annotated in pineapple (**Supplementary Table S1**). Two maximum likelihood trees (ML) were further constructed on the basis of the full-length sequence alignment of all *AcMADS* genes together with grape and *Arabidopsis* to provide a reference for the evolutionary relationship of the MADS-box family in pineapple (**Figure 2**). The 44 *AcMADS* genes of the pineapple can be divided into two categories, namely, type I (12) and type II (32). In the first ML tree, type I *AcMADS* genes were further classified into three subclasses: Mα, Mβ, and Mγ (**Figure 2A**). One MIKC*-type and 27 MIKC^C^-type genes were showed in the second ML tree, and the MIKC^C^-type genes were further classed into 11 major groups (**Figure 2B**). These groups were named after the *Arabidopsis* genes as follows: *SVP* (*SHORT VEGETATIVE PHASE*), *AGL12*, *SEP*, *AGL6*, *AP1*, *FLC* (*FLOWERING LOCUS C*), *SOC1* (S*UPPRESSOR OF OVEREXPRESSION OF CONSTANS1*), *AG*, *PI/AP3*, *BS* (*Bsister*), and *ANR*.

### Chromosomal location and duplication analysis of pineapple MADS-box genes

**Figure 3:**
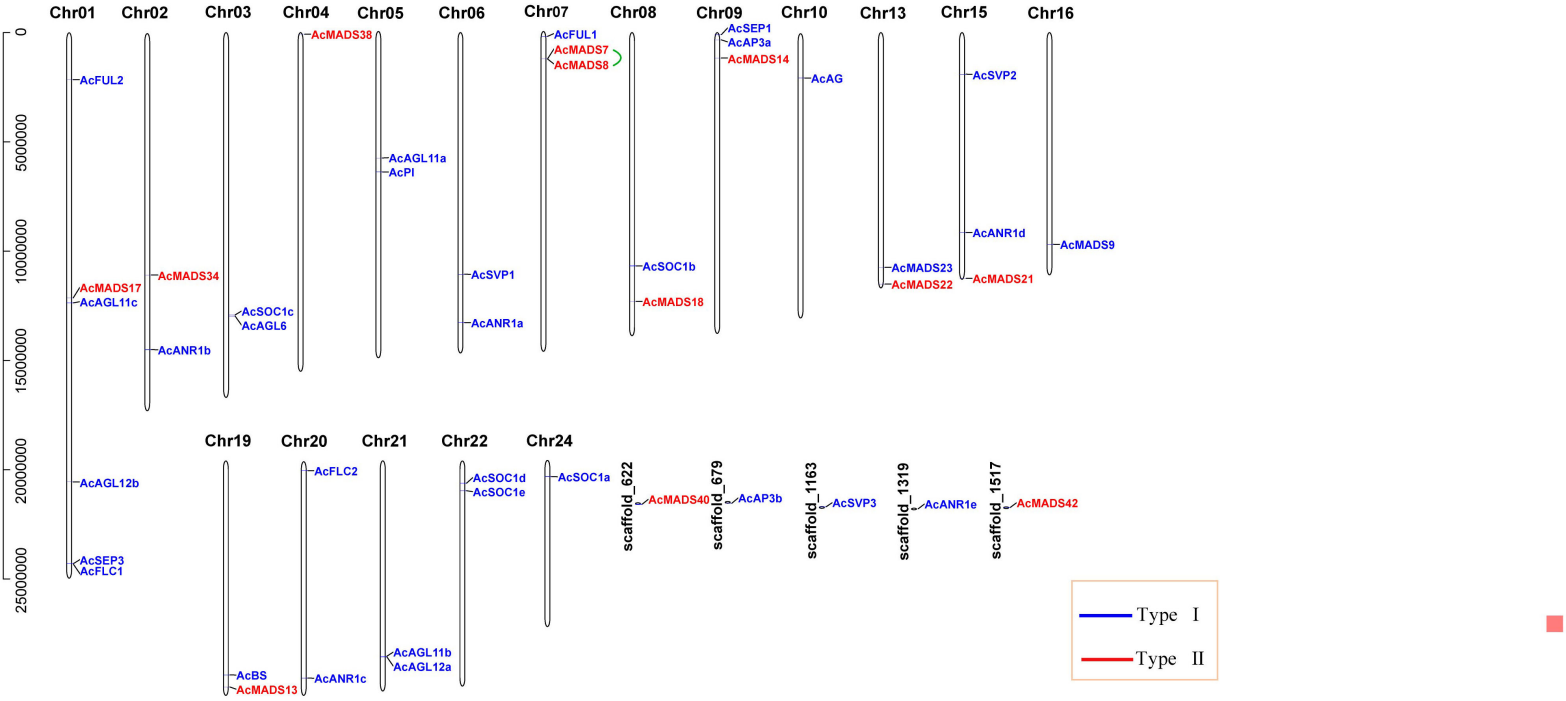
Distribution of *AcMADS* genes on the pineapple chromosomes. The black vertical lines indicate the pineapple chromosome (Chr), the number is above the Chr, and the tandemly duplicated genes were connected by the green line. The different groups of *AcMADS* genes were color-coded.

Forty-four MADS-box genes were unevenly mapped to the 18 chromosomes (Chr) and five scaffolds and named from *AcFUL2* to *AcMADS42* according to their order on the chromosomes. The largest number of six genes (13.64%) was found in Chr01, and the other chromosomes contained less than three *AcMADS* genes. Among the 44 *AcMADS* genes, 12 genes of type I were distributed on 10 chromosomes and three scaffolds, and 32 genes of type II were mapped to 17 chromosomes and 3 scaffolds. Chr04 only contained type I genes, while Chr03, Chr05, Chr06, Chr10, Chr16, Chr20, Chr21, Chr22, and Chr24 only had type II genes (**Figure 3**). Tandem duplication event refers to a region of chromosomes within 200 kb containing two or more genes (Holub, 2001). The gene replication events of the MADS-box family found that one pair of genes (*AcMADS7*/*AcMADS8*) underwent tandem repeat events within the *AcMADS* gene family on Chr7 (**Figure 3**). These results showed randomness and nonuniform distribution of the *AcMADS* family in pineapple.

### Syntenic analysis of pineapple *AcMADS* genes

**Figure 4:**
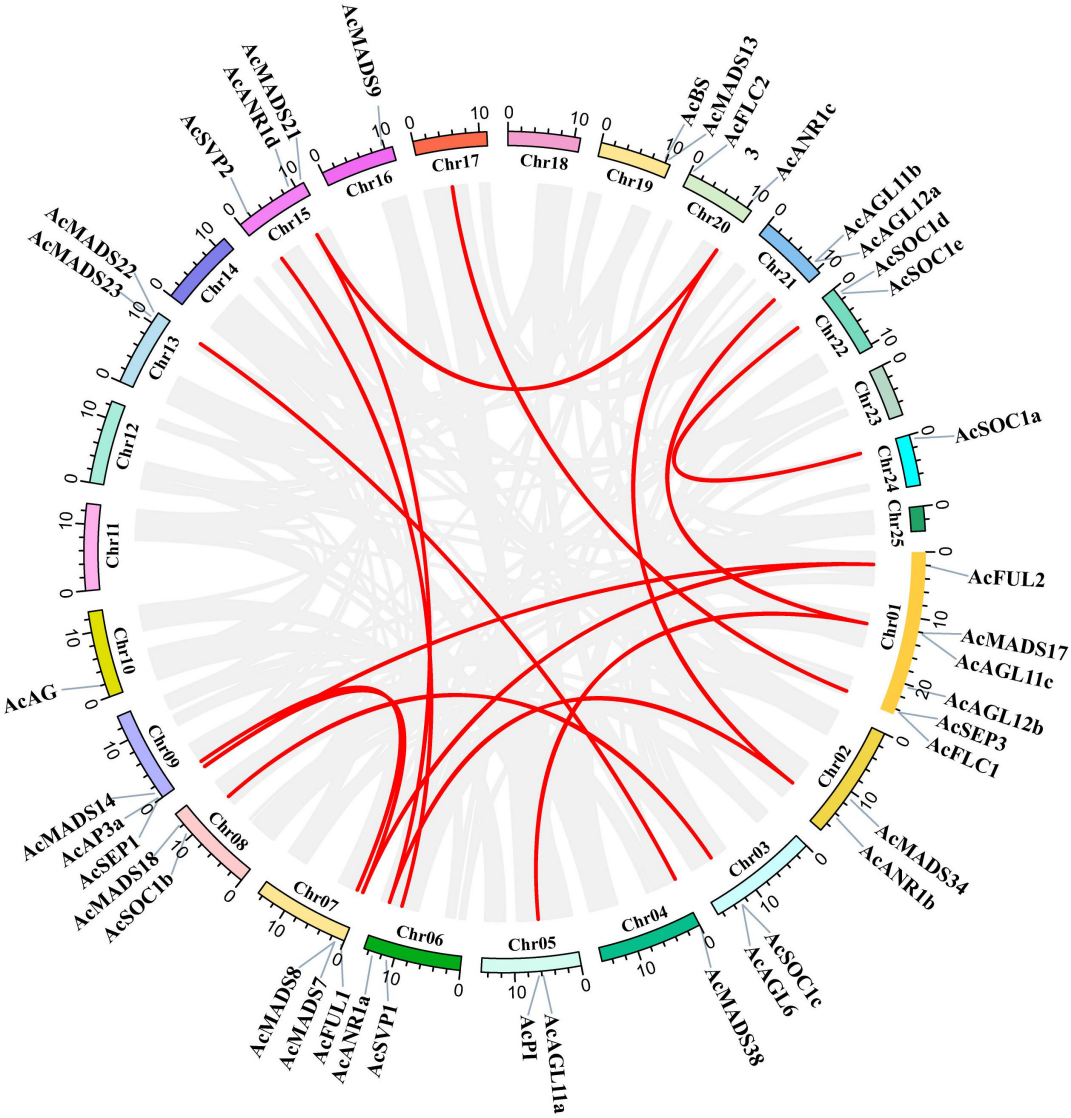
Schematic representations for interchromosomal relationships and segmental duplication events of pineapple MADS genes. The gray lines represent all collinear blocks within the pineapple genome, and the red lines denote duplicated MADS gene pairs.

The segmental and tandem duplication events of the *AcMADS* genes family were identified to test the duplication effect in pineapple. In addition to the above-mentioned two tandem duplication events, twenty-two *AcMADS* genes were clustered into 15 segmental duplication events by BLASTP and MCScanX methods against the published data of the pineapple genome (http://pineapple.angiosperms.org/pineapple/html/index.html). And the pineapple had retained a diploid karyotype in this study (**Figure 4**, **Supplementary Table S2**). We found many copies of the segmental duplicated gene pairs from the same group, such as *AcANR1a*/*AcANR1b*. *AcANR1b*/*AcANR1c* and *AcSVP1*/*AcSVP*2 were from the ANR and SVP groups, respectively. *AcAGL11a*/*AcAGL11c* and *AcAGL11b*/*AcAGL11c* were from the AG group, *AcFUL1*/*AcFUL2* were from the AP1 group, and *AcSOC1a*/*AcSOC1e* were from SOC1. These *AcMADS* genes showed highly paralogs relationship, thereby indicating that they were obtained by gene duplication and the segmental duplication could well be the main driving force of *AcMADS* evolution.

**Figure 5:**
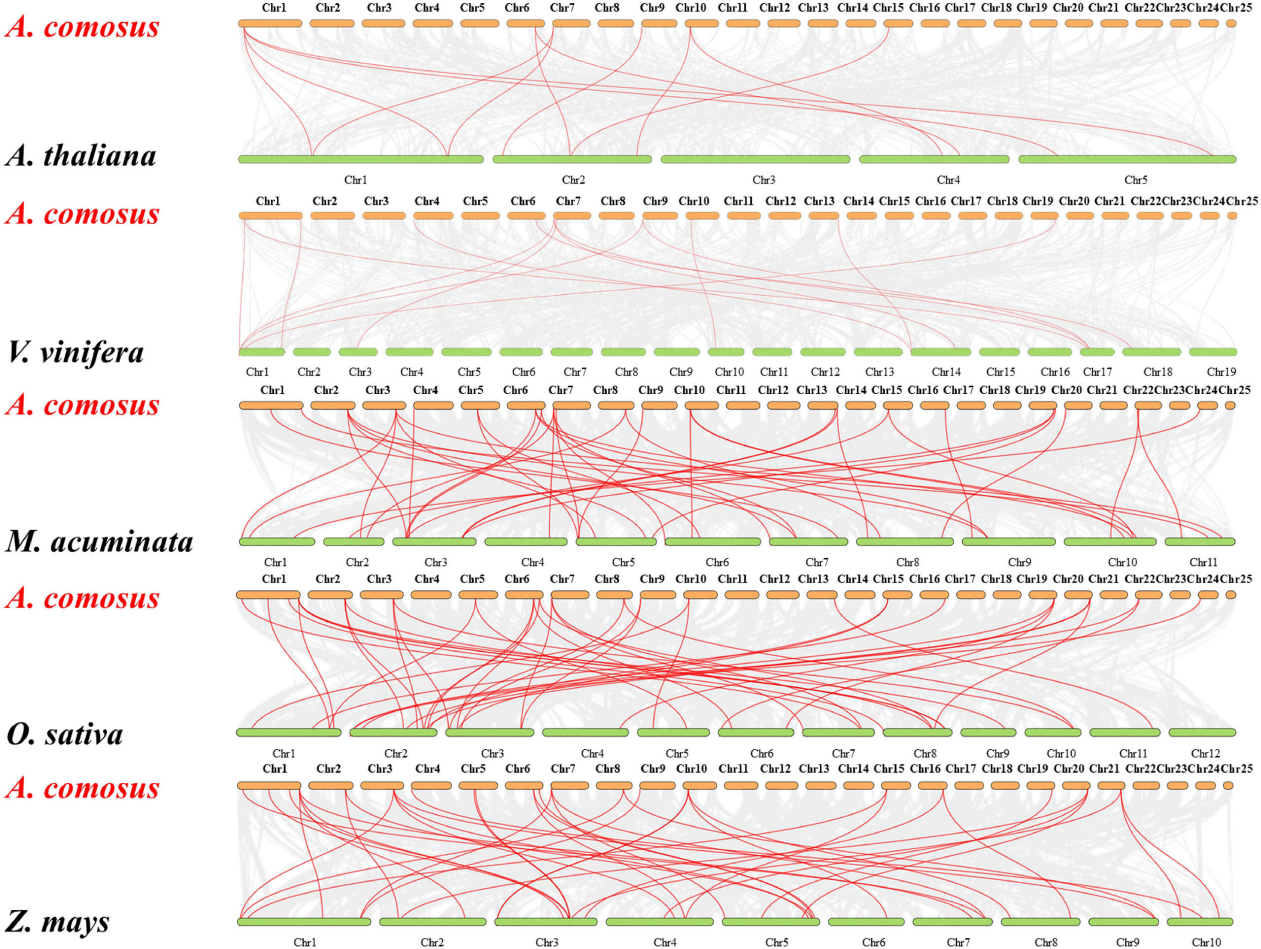
Syntenic analysis of MADS-box genes between pineapple and other five species. The gray lines represent the collinear blocks between pineapple and other plant genomes. The red lines indicate the collinear blocks of the pineapple MADS genes. Species names: A. comosus: *Ananas comosus*, A. thaliana: *Arabidopsis thaliana*, V. vinifera: *Vitis vinifera*, M. acuminate: *Musa acuminate*, O. sativa: *Oryza sativa*, Z. mays: *Zea mays*.

The comparative syntenic maps between pineapple with other five representative species (*Arabidopsis*, grape, banana, rice, and maize) were performed to further derive the origin and evolutionary mechanisms of pineapple MADS family (**Figure 5**). The gene number of pineapple MADS (44) is much lower than that of *Arabidopsis* (106), banana (77), rice (75), maize (75), and grape (54). Twenty-four *AcMADS* genes displayed syntenic relationship with those in banana, followed by maize (23, 52%), rice (22, 50%), grape (10, 32%), and *Arabidopsis* (6, 13%) through the whole genome-wide comparative analysis. The numbers of orthologous pairs between pineapple and other five species (banana, rice, maize, grape, and *Arabidopsis*) were 42, 42, 40, 14, and 12, respectively (**Supplementary Table S3**). Many collinear gene pairs were only found in monocots but not in dicots, thereby suggesting evolutionary difference between dicotyledonous and monocotyledonous plants. The mutual collinear pairs involving three *AcMADS* genes were identified between pineapple and all five other species. This finding indicates that these orthologous pairs may be derived from the same ancestor, and duplication occurred before species divergence. These pairs may participate in the evolution of MADS family.

### Analysis of *cis*-regulatory elements in the promoters of *AcMADS* genes

**Figure 6:**
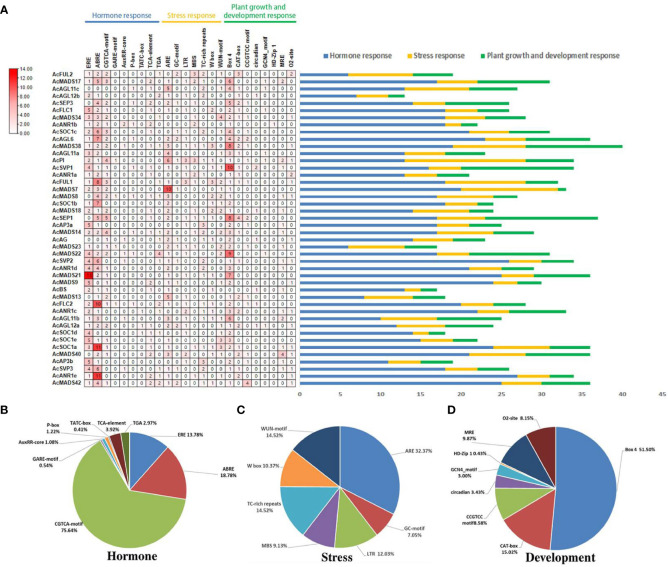
Organization of *cis*-acting regulatory elements of *AcMADS* genes in pineapple. **(A)** The number of *cis*-acting elements of AcMADS genes. **(B)** The total amount of each *cis*-acting elements as a percentage of hormone-responsive elements. **(C)** The total amount of each *cis*-acting elements as a percentage of stresses-related elements. **(D)** The total amount of each *cis*-acting elements as a percentage of the cis-acting element involved in plant development and growth.

To explore the regulatory mechanisms of *AcMADS* genes, 24 types of *cis*-elements in the promoter sequence of *AcMADS* genes were investigated and compared with the PlantCare online database (**Figure 6**). Among the 24 types of *cis*-acting elements, three kinds of regulatory elements of core physiological processes including abiotic stress, hormone responsive and growth related were investigated (**Figure 6**).

Among them, nine hormone-responsive elements were found in *AcMADS* genes including Auxin-related elements (TGA-element and AuxRR-core), the *cis*-acting element involved in Sali-cylic Acid responsiveness (TCA-element), Gibberellin responsive elements (TATC-box, P-box and GARE-motif) MeJA-related element (CGTCA-motif), Abscisic Acid (ABRE) and Ethylene (ERE). The ERE and ABRE elements have a wide distribution in *AcMADS* genes, which were identified in 37 (84%) and 39 (88.6%) *AcMADS* genes, among which the sequences of *AcANR1d* and *AcSVP2* contained 13 ERE and 11 ABRE elements, respectively (**Figure 6**). These results indicated that *AcMADS* genes might be related to ethylene and abscisic acid signaling pathway.

The *cis*-acting element involved in plant development and growth, for instance, GCN4_motif, CAT-box, and CCGTCC-box, which they were involved in albumen development and meristem expression. Furthermore, the elements are included light responsive elements (BOX4 and MRE), circadian control responsiveness (circadian), protein metabolism regulation (O2-site), and the differentiation of the palisade mesophyll cells (HD-Zip 1). About half of the 88.6% *AcMADS* genes have Box4 elements, and the sequences of *AcAGL6*, *AcSOC1b*, *AcAG*, *AcANR1d*, *AcMADS21*, *AcSOC1d* and *AcANR1e* promoter distributed plenty of Box4 elements (**Figure 6**). These results provide a reference for further study of *AcMADS* family genes in flower organ differentiation of pineapple.

The abiotic stresses-related elements, including ARE, GC-motif, LTR (low temperature), MBS (drought), TC-rich repeat, W-box and WUN-motif. In pineapple, 35 (79.5%) *AcMADS* genes have ARE elements, less more than Box4 and ERE, and 20.4% of *AcMADS* promoter sequence contained 3 or more ARE elements (**Figure 6**). Among which, the amount of *AcANR1a*, *AcMADS18*, *AcMADS14* and *AcAP3b* are up to 10, 5, 5 and 6 (**Figure 6**).

### Tissue-specific expression patterns of *AcMADS* genes in pineapple

**Figure 7:**
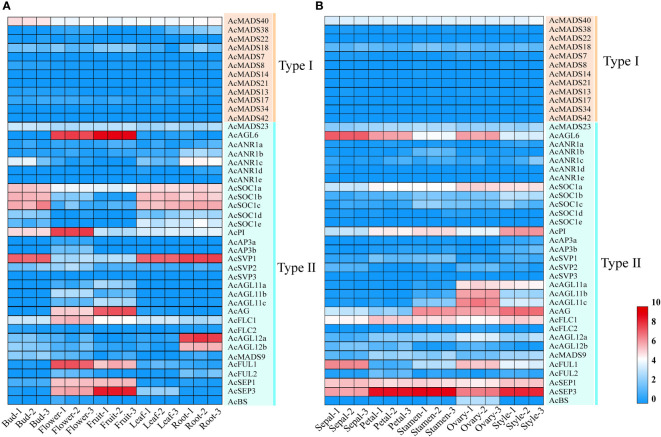
Expression patterns of the pineapple MADS genes, the transcript abundance of *AcMADSs* was computed by FPKM values. **(A)** Hierarchical clustering of the expression patterns of *AcMADS* genes in the different tissues of pineapple. **(B)** Expression profiles of *AcMADS* genes in pineapple floral organs.

MADS-box genes were reported to participate in plant organ development, especially floral organ specification (Saedler et al., 2001). The expression patterns of 44 pineapple *AcMADS* genes in different tissues were obtained from the transcriptome data. The results showed that the transcriptional abundance of MADS genes in pineapple greatly varied in all detected samples (**Figure 7**). The genes with high expression were mainly concentrated in type II subfamily. Twenty-six *AcMADS* genes (59%) were highly expressed in the flower of pineapple. Meanwhile, 14 *AcMADS* genes (32%) were highly expressed in the fruit of pineapple. Sixteen genes (36%) were expressed at low levels or not expressed in pineapple root, bud, leaf, flower, and fruit (**Supplementary Table S4**). Multiple *AcMADS* genes were specifically expressed in flower and fruit. For example, five *AcMADS* genes (*AcSEP1*/*AcSEP3*/*AcAGL6*/*AcFUL1*/*AcAG*) showed high transcript abundance in flower and fruit, two genes (*AcFLC1*/*AcPI*) only demonstrated high transcript abundance in flowers, and two genes (*AcAGL12a*/*AcAGL12b*) only presented high expression in the roots (**Figure 7A**). These results implied that MADS-box genes might participate in the flower development of pineapple, which was in line with previous reports (Cheng et al., 2017).

**Figure 8:**
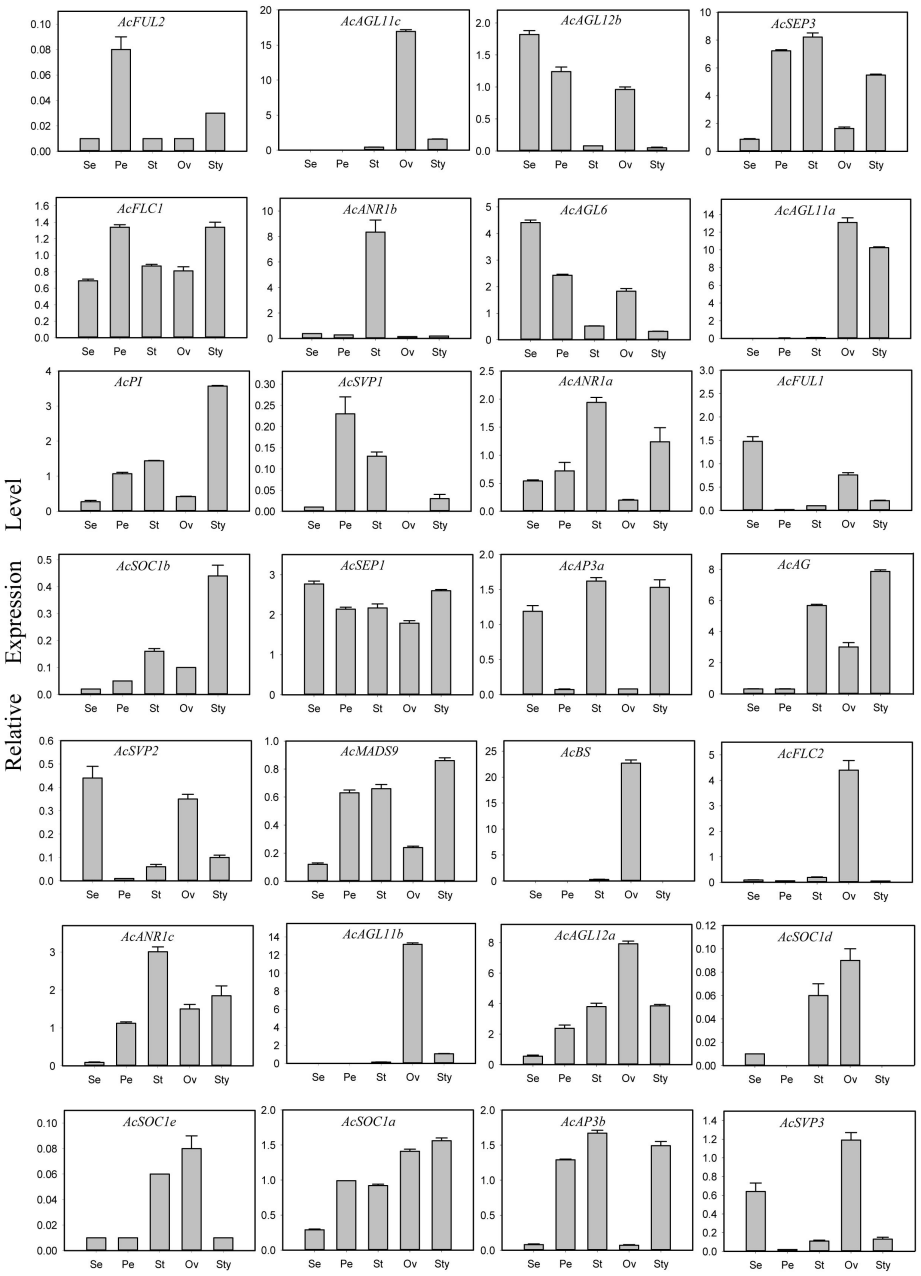
Expression patterns of 28 type II *AcMADS* genes in pineapple floral organs by qRT-PCR. The error bars represent the standard deviations of three biological replicates. Se: Sepal, Pe: Petals, St: Stamen, Ov: ovary, and Sty: Style.

**Figure 9:**
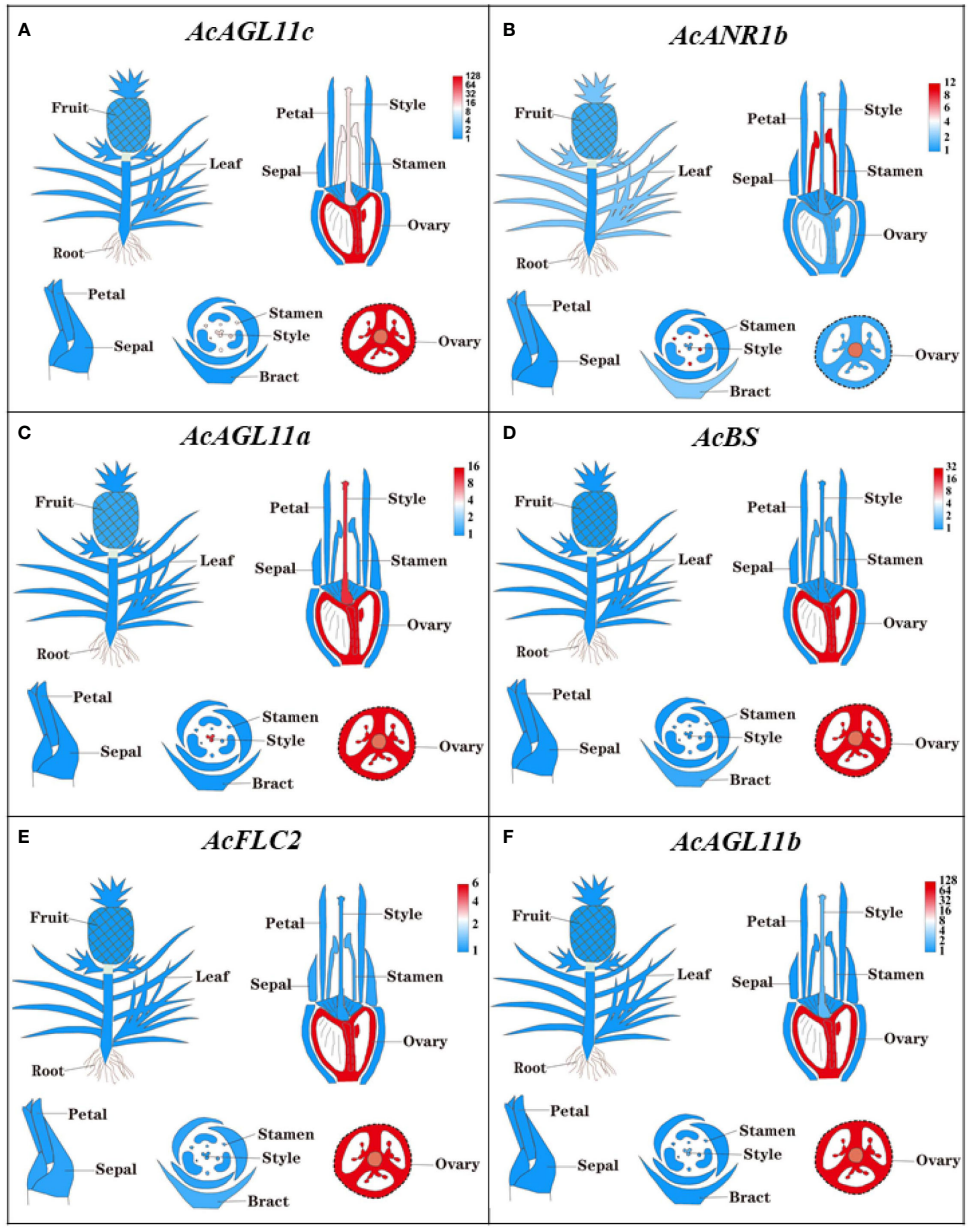
The expression of AcMADS genes in different floral organs by TBtools. The transcript abundance of AcMADSs were computed by FPKM values and visualized by TBtools. Red module represents tissue high expression and blue module showed low expression.

The expressions of all 44 *AcMADS* genes in different floral organs were investigated to further verify the potential functions of the *AcMADS* genes in the formation of floral organs (**Figure 7B**). Thirty-eight *AcMADS* genes exhibited relatively high expression in one or more flower organs of pineapple, and fifteen of them were highly expressed. Most *AcMADS* genes with high expression in floral organs originated from the type II subfamily, thereby suggesting that *AcMADS* genes of type II may play crucial roles in the development of floral organs of pineapple contrast with type I (**Figure 7B**). Twenty-eight type II *AcMADS* genes were further performed by qRT-PCR to validate the RNA-seq results (**Figure 8**). The qRT-PCR results showed that the expression profiles of most *AcMADS* genes were consistent with RNA-Seq. Many *AcMADS* genes showed obvious tissue specificity. For example, *AcAGL11a* only expressed in pistil, *AcAGL11c*, *AcBS*, *AcFLC2*, and *AcAGL11b* had specifically high expression in the ovary, and *AcANR1b* only expressed in the stamen (**Figure 9**).

### Interaction network of key *AcMADS* proteins

**Figure 10:**
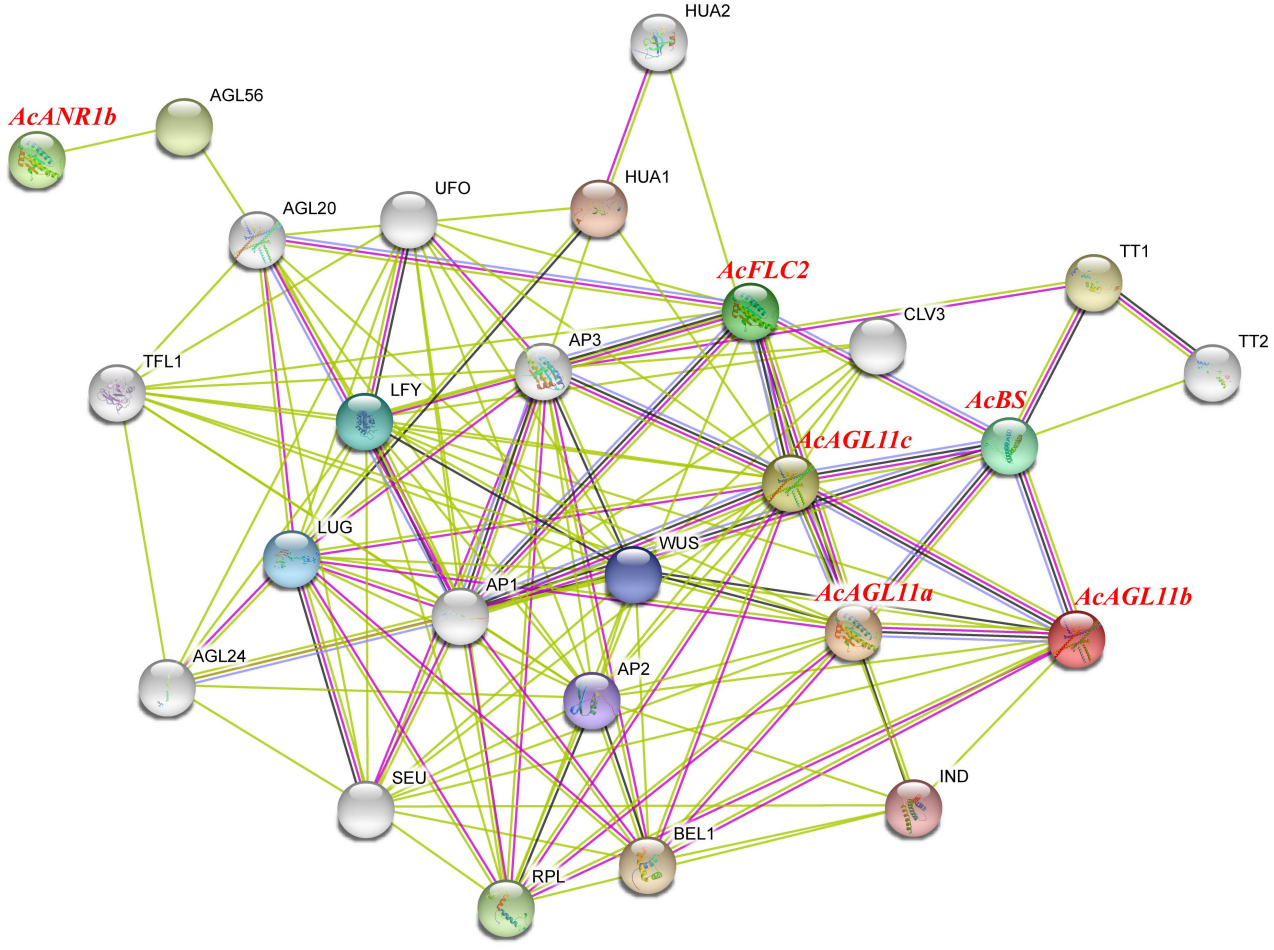
Functional interaction network between *AcMADS* proteins and other related proteins according to the orthologs in *Arabidopsis*. Pink line: experimentally determined; green line: gene neighborhood; red line: gene fusions; blue line: gene co-occurrence; cyan line: text mining; black line: co-expression.

Plant flowering is a complex physiological process that requires multiple genes to work together. Studying protein interaction network contributes to the exploration of the potential functions of genes. The qRT-PCR and RNA-seq results indicated that six *AcMADS* genes with specific expressions in the floral organs of pineapple, and they were selected to construct an interaction network through the String Protein Interaction Database (https://string-db.org/). A total of 129 protein pairs with interactions were detected. These interaction proteins were mainly involved in the floral organ developmental genes and floral induction, including AP1, CO, WUS, SEU, AP2, UFO, and LFY (**Figure 10**). In the protein interaction network diagram, AcAGL11c interacted with 10 known proteins with the largest number of interacting proteins. AcAGL11b, AcAGL11a, AcBS, and AcFLC2 interacted with 2, 4, 6, and 8 known proteins, respectively. AcANR1b only interacted with one known protein. These results will be beneficial to future research and verify its biological function on the basis of relevant experiments.

## Discussion

The MADS-box genes play crucial roles in the development of floral organs and had been investigated in multiple species (Parenicová et al., 2003; Leseberg et al., 2006; Tyagi et al., 2007; Velasco et al., 2010; Wang et al., 2014). However, previous studies only analyzed some functions of *AcMADS* genes in the CAM photosynthesis of pineapple (Zhang et al., 2020). In this study, 44 genes with typical MADS domains were defined as MADS-box family genes, which is inconsistent with previous research (Zhang et al., 2020; Hu et al., 2021). The number of MADS-box in pineapple (44) is much lower than that of *Arabidopsis* (106) (Parenicová et al., 2003), rice (75) (Tyagi et al., 2007), poplar (105) (Leseberg et al., 2006), apple (147) (Velasco et al., 2010), and grape (54) (Wang et al., 2014). The lack of pan-grass ρ whole-genome duplication (WGD) event and only having the σ-WGD event during pineapple evolution may affect the amount of *AcMADS* genes (Xie et al., 2018).

The *AcMADS* family of pineapple is classified into two categories, namely, types I and II; the type II genes are further divided into 11 subfamilies (**Figure 2B**). However, the classification and number of type II *AcMADS* are slightly different among different species. The genome-wide duplication events are common in angiosperm evolution and generally result in the expansion of gene families (Cannon et al., 2004). Type II MADS genes originate from the whole genome replication, while type I genes are mainly duplicated by small-scale and recent duplications (Airoldi and Davies, 2012). In our studies, 22 of the 44 MADS-box genes are associated with segmental duplication events, which are higher than that of the *Arabidopsis* (Parenicová et al., 2003). The exploration of the genes related to flower development and flowering remains to be a major research topic. MADS-box genes play a major role in determining the identity of floral organs (Li et al., 2020), and most type II genes are extensively expressed in the reproductive organs and lowly expressed in vegetative organs (Sheng et al., 2019). In this study, 38 *AcMADS* genes are expressed in one or more tissues, and 13 of them are specially expressed in flowers. The type II of *AcMADS* genes are highly expressed compared with type I in flower organs (**Figure 7B**), thereby indicating that the type II MADS genes might play more important roles in the flower development of pineapple. This finding is consistent with previous reports (Li et al., 2020). The ABCDE model is a classical model of plant flower development (Kanno et al., 2003; Litt and Kramer, 2010). In *Arabidopsis*, *AP1* acts as a gene for flower meristem and organ morphology to promote the development of petals and sepals (Ma, 2000; Portereiko et al., 2006). Besides, *OsMADS15* and *OsMADS18* were activated in the rice meristem at phase transition (Kobayashi et al., 2012). In pineapple, two homologous genes of class-A (*AcFUL2* and *AcFUL1*) are identified. The expression of *AcFUL2* and *AcFUL1* was notably higher in petals and sepals than in other floral tissues, respectively (**Figure 8**), which is in line with its expected function in sepal identity specification. Our results showed that two AP1-like genes were uniformly expressed in pineapple floral organs. This similar expression pattern in floral organs was also for AP1-like genes in *Arabidopsis* (Mandel et al., 1992) and rice (Arora et al., 2007). The main function of class-B genes (*AP3* and *PI*) is to determine the development of the second round of petals and the third round of stamens in *Arabidopsis* (Kanno et al., 2003). Two AP3-like (*AcAP3b* and *AcAP3a*) and one PI-like (*AcPI*) genes from the class-B genes are identified in pineapple and have similar expression patterns (**Figure 8**). Rice *OsMADS16*/*SPW1* and maize *SILKY1* (*SL*) mRNA were detected mainly in the stamen during floral development, besides the expression of *TaAP3* was obviously accumulated in mature female organs (Paollacci et al., 2007). For pineapple, *AP3a* and *AP3b* show a similar expression pattern: mainly in stamen and style development, whether *AcPI* was highly expressed only in style (**Figure 8**).The expression features of class-B genes in pineapple are similar to those of *Arabidopsis*, which participate in the second and third rounds of floral organ formation. Additionally, these genes in pineapple may also be involved in the formation of the fourth round of pistil.

*AG* is a typical class-C gene and essential for the identification of stamens and carpels (Pinyopich et al., 2003). Four *AcMADS* genes of class-C were detected in pineapple (**Figure 2B**). *AcAGL11c*, *AcAGL11a*, and *AcAGL11b* are specifically highly expressed in the pistil, and *AcAG* shows high expression in the stamen and pistil (**Figure 8**). *AcAGL11c* and *AcAGL11a*, *AcAGL11c* and *AcAGL11b* are also involved into the segment replication events (**Figure 4**). These results show that these genes of class-C might play similar roles in the development of stamen and pistil of pineapple. The homologous genes of *AG* are involved in the development of stamen and carpel in pineapple, which was consistent with previous reports (Pelaz et al., 2000). In *Arabidopsis*, the SEP proteins are functionally important to form higher MADS-box protein complexes (Acri-Nunes-Miranda and Mondragón-Palomino, 2014; Favaro et al., 2003). Genetic and molecular studies have shown that class-E genes (*SEP1/2/3/4*) have an obvious redundant function in flower development and are necessary to determine all four whorls of the flower organs (Favaro et al., 2003; Acri-Nunes-Miranda and Mondragón-Palomino, 2014). In pineapple, two SEP-like genes (*AcSEP1* and *AcSEP3*) were identified (**Figure 2B**). AcSEP3 was highly expressed throughout the petal, stamen and style, and AcSEP1 was highly expressed in all floral organs (**Figure 8**). AcSEP3 is more likely to have an important role in class-E function because it had higher expressions than AcSEP1 in all organs except sepals. Additionally, *AcSEP3* homologs *AtAGL9* and *OsMADS7*/*8* also play relatively important roles in *Arabidopsis* and rice (Soza et al., 2016; Wu et al., 2017) (**Figure 7B**). By comparing the expression patterns of MADS genes in pineapple and the functions of their previously reported homologs, we inferred candidate MADS genes in pineapple that may be involved in floral organ development.

Six genes have significantly specific expression in one or two floral organs of pineapple (**Figure 9**). *AcAGL11c*, *AcAGL11a*, and *AcAGL11b* belong to the AG subgroup, while *AcANR1b*, *AcBS*, and *AcFLC2* belong to the *ANR*, *BS*, and FLC subgroups (**Figure 2B**). *AcANR1b* is specifically expressed in the stamens; however, homologous gene *AtAGL16*, which negatively regulates flowering transition through *FLOWERING LOCUS T* (*FT*), is not found in stamens (Hu et al., 2014). This finding indicates that *AcANR1b* could play a novel and key role in the development of pineapple stamens. *BS* (*AT5G23260.2*) might be involved in the developmental regulation of the endothelium, which may be essential for ovule development (Xu et al., 2017). *AcBS*, a homologous gene of *AtBS*, is specifically expressed in the ovary of pineapple, manifested that *AcBS* might be involved in the development of ovary, and may be essential for the formation of female gametophyte of pineapple. *AcFLC2*, which is homologous with FLC is a flowering repressor in *Arabidopsis* (Richter et al., 2019), is also specifically expressed in the ovary of pineapple. However, the involvement of *AcFLC2* gene in pistil development is yet to be reported. These *AcMADS* genes could directly or indirectly determine the formation of pineapple floral organs and provide a reference for further exploring the molecular mechanism of pineapple flower formation, but their potential functions still need to be further verified by systematic experiments.

## Conclusions

In this study, the evolution and functional differentiation of the MADS-box genes of pineapple were comprehensively analyzed, and the expression profile of the *AcMADS* genes in the floral organ were proposed. The type II *AcMADS* genes played crucial roles in the development of floral organs of pineapple. *AcAGL11c*/*AcAGL11a*/*AcAGL11b* of the AG subfamily and *AcBS* of the TTI6 subfamily were highly related to ovary development and pistil formation. *AcANR1b* of the ANR subfamily controlled the formation of stamens. Thus, these genes can be identified as candidate genes for vector construction and further functional analysis. These genes provided resources for exploring the regulation network of pineapple flowering and references for the genetic improvement of transgenic crops and traditional breeding.

## Data availability statement

The datasets presented in this study can be found in online repositories. The names of the repository/repositories and accession number(s) can be found in the article/**Supplementary Material**. The transcriptome data have uploaded into National Genomics Data Center database, and the assigned accession of the submission is: CRA006826. Sequence data used in the study can be found in **Supplementary Material**.

## Author contributions

XP wrote the manuscript. HZ designed the experiment and contributed to data. YO took charge the experimental materials treatment and collection. YW investigated the data analyze of the study. BZ and JW carried out RNA extraction and Q-PCR verification. HZ conceived the study. All authors contributed to the article and approved the submitted version.

## Funding

The project was funded by the National Key R&D Program of China (2019YFD1001105 and 2018YFD1000504), the National Natural Science Fund of China (31872079 and 32160687), the Natural Science Foundation of Hainan Province (321RC467 and 322MS013), the major science and technology project of Hainan Province (ZDKJ2021014), and the Scientific Research Start-up Fund Project of Hainan University (KYQD-ZR-20090).

## Conflict of interest

The authors declare that the research was conducted in the absence of any commercial or financial relationships that could be construed as a potential conflict of interest.

## Publisher’s note

All claims expressed in this article are solely those of the authors and do not necessarily represent those of their affiliated organizations, or those of the publisher, the editors and the reviewers. Any product that may be evaluated in this article, or claim that may be made by its manufacturer, is not guaranteed or endorsed by the publisher.
