# Supplementary material for: Genome-wide analysis of MADS-box families and their expressions in flower organs development of pineapple (Ananas comosus (L.) Merr.)
Source: Front Plant Sci. 2022 Oct 12;13:948587. doi: 10.3389/fpls.2022.948587 (PMC9597317; doi:10.3389/fpls.2022.948587)
Supplement: Supplementary file 1 [file Table_1.docx]

**Table S1.** Pineapple MADS-box genes

| **Gene Name** | **Gene ID** | **Family** | | **ORF length（bp）** | **Size（aa）** | **MW(Da)** | **pI** | **Predicted Subcellular location** | **Chromosome No.** | **Group** |
| --- | --- | --- | --- | --- | --- | --- | --- | --- | --- | --- |
| AcFUL2 | Aco012428.1 | MIKC_MADS | | 1074 | 358 | 39441.4 | 8.2907 | Nuclear | LG1 | AP1/FUL |
| AcMADS17 | Aco011374.1 | M-type_MADS | | 1263 | 421 | 49214.8 | 10.4235 | Nuclear | LG1 | Mβ |
| AcAGL11c | Aco011341.1 | MIKC_MADS | | 705 | 235 | 26960.5 | 9.6899 | Nuclear | LG1 | AGL11 |
| AcAGL12b | Aco018015.1 | MIKC_MADS | | 645 | 215 | 24368.1 | 6.8541 | Nuclear | LG1 | AGL12 |
| AcSEP3 | Aco015105.1 | MIKC_MADS | | 741 | 247 | 28217.2 | 8.2885 | Nuclear | LG1 | SEP |
| AcFLC1 | Aco015104.1 | MIKC_MADS | | 759 | 253 | 27924.9 | 9.0267 | Nuclear | LG1 | FLC |
| AcMADS34 | Aco019026.1 | M-type_MADS | | 288 | 96 | 10838.7 | 8.766 | Nuclear | LG2 | Mβ |
| AcANR1b | Aco001069.1 | MIKC_MADS | | 882 | 294 | 33676.5 | 9.9661 | Nuclear | LG2 | ANR1 |
| AcSOC1c | Aco015492.1 | MIKC_MADS | | 492 | 164 | 18661.9 | 11.7942 | Nuclear | LG3 | SOC1 |
| AcAGL6 | Aco015487.1 | MIKC_MADS | | 726 | 242 | 27527.3 | 9.5947 | Nuclear | LG3 | AcAGL6 |
| AcMADS38 | Aco022101.1 | M-type_MADS | | 804 | 268 | 29595.1 | 8.469 | Nuclear | LG4 | Mγ |
| AcAGL11a | Aco004785.1 | MIKC_MADS | | 717 | 239 | 27267.8 | 9.5516 | Nuclear | LG5 | AGL11 |
| AcPI | Aco019365.1 | MIKC_MADS | | 594 | 198 | 22876.4 | 9.8523 | Nuclear | LG5 | PI |
| AcSVP1 | Aco002729.1 | MIKC_MADS | | 897 | 299 | 33321.3 | 9.7367 | Nuclear | LG6 | SVP |
| AcANR1a | Aco003018.1 | MIKC_MADS | | 744 | 248 | 28006.8 | 7.5387 | Nuclear | LG6 | ANR1 |
| AcFUL1 | Aco004839.1 | MIKC_MADS | | 747 | 249 | 28426.4 | 9.6366 | Nuclear | LG7 | AP1/FUL |
| AcMADS7 | Aco004987.1 | M-type_MADS | | 672 | 224 | 24161.5 | 9.63 | Nuclear | LG7 | Mα |
| AcMADS8 | Aco004988.1 | M-type_MADS | | 1311 | 437 | 45315.6 | 7.9304 | Nuclear | LG7 | Mα |
| AcSOC1b | Aco016643.1 | MIKC_MADS | | 627 | 209 | 24006.7 | 9.4595 | Nuclear | LG8 | SOC1 |
| AcMADS18 | Aco011677.1 | M-type_MADS | | 573 | 191 | 21248.8 | 6.7979 | Nuclear | LG8 | Mα |
| AcSEP1 | Aco017563.1 | MIKC_MADS | | 744 | 248 | 28158 | 8.7473 | Nuclear | LG9 | SEP |
| AcAP3a | Aco017589.1 | MIKC_MADS | | 468 | 156 | 18242.9 | 10.0693 | Nuclear | LG9 | APETALA3 |
| AcMADS14 | Aco008623.1 | M-type_MADS | | 687 | 229 | 24657.9 | 7.5115 | Nuclear | LG9 | Mα |
| AcAG | Aco009993.1 | MIKC_MADS | | 762 | 254 | 29600.6 | 9.6409 | Nuclear | LG10 | AG |
| AcMADS23 | Aco013736.1 | MIKC_MADS | | 1053 | 351 | 39543.4 | 5.8089 | Nuclear | LG13 | MIKC* |
| AcMADS22 | Aco013644.1 | M-type_MADS | | 762 | 254 | 28251.4 | 9.8024 | Nuclear | LG13 | Mγ |
| AcSVP2 | Aco004028.1 | MIKC_MADS | | 693 | 231 | 25957.6 | 8.7072 | Nuclear | LG15 | SVP |
| AcANR1d | Aco019842.1 | MIKC_MADS | | 420 | 140 | 15129.4 | 10.5837 | Nuclear | LG15 | ANR1 |
| AcMADS21 | Aco013324.1 | M-type_MADS | | 717 | 239 | 25850.9 | 8.6411 | Nuclear | LG15 | Mα |
| AcMADS9 | Aco006017.1 | MIKC_MADS | | 591 | 197 | 22878.2 | 8.2291 | Nuclear | LG16 | AGL12 |
| AcBS | Aco008359.1 | MIKC_MADS | | 720 | 240 | 27535.3 | 7.7216 | Nuclear | LG19 | Bsister |
| AcMADS13 | Aco008435.1 | M-type_MADS | | 996 | 332 | 35788.8 | 7.4737 | Nuclear | LG19 | Mα |
| AcFLC2 | Aco019039.1 | MIKC_MADS | | 630 | 210 | 23137.6 | 10.3109 | Nuclear | LG20 | FLC |
| AcANR1c | Aco014671.1 | MIKC_MADS | | 711 | 237 | 26952.8 | 8.6563 | Nuclear | LG20 | ANR1 |
| AcAGL11b | Aco007999.1 | MIKC_MADS | | 702 | 234 | 26894.7 | 9.9592 | Nuclear | LG21 | AGL11 |
| AcAGL12a | Aco007995.1 | MIKC_MADS | | 552 | 184 | 20772.8 | 5.8619 | Nuclear | LG21 | AGL12 |
| AcSOC1d | Aco030142.1 | MIKC_MADS | | 525 | 175 | 19557.3 | 7.4455 | Nuclear | LG22 | SOC1 |
| AcSOC1e | Aco017499.1 | MIKC_MADS | | 606 | 202 | 23368.7 | 9.1528 | Nuclear | LG22 | SOC1 |
| AcSOC1a | Aco013229.1 | MIKC_MADS | | 639 | 213 | 24130.7 | 9.5311 | Nuclear | LG24 | SOC1 |
| AcMADS40 | Aco027629.1 | M-type_MADS | | 4569 | 1523 | 166541 | 4.5399 | Chloroplast |  | Mγ |
| AcAP3b | Aco025594.1 | MIKC_MADS | | 468 | 156 | 18242.9 | 10.0693 | Chloroplast |  | APETALA3 |
| AcSVP3 | Aco027879.1 | MIKC_MADS | | 693 | 231 | 25899.6 | 9.1243 | Chloroplast |  | SVP |
| AcANR1e | Aco030553.1 | MIKC_MADS | | 180 | 60 | 6676.85 | 11.327 | Chloroplast |  | ANR1 |
| AcMADS42 | Aco028086.1 | M-type_MADS | | 1056 | 352 | 39808.5 | 4.7424 | Chloroplast |  | Mβ |
|  |  |  |  | |  |  |  |  |  |  |
