# Supplementary material for: Genome-wide analysis of MADS-box families and their expressions in flower organs development of pineapple (Ananas comosus (L.) Merr.)
Source: Front Plant Sci. 2022 Oct 12;13:948587. doi: 10.3389/fpls.2022.948587 (PMC9597317; doi:10.3389/fpls.2022.948587)
Supplement: Supplementary file 2 [file Table_2.docx]

**Table S2.** Synteny blocks of *AcMADS* genes within pineapple genome

| **Chr name** | **Gene name** | **Chr name** | | **Gene name** |
| --- | --- | --- | --- | --- |
| LG1 | Aco011341.1 | | LG5 | Aco004785.1 |
| LG1 | Aco012428.1 | | LG7 | Aco004839.1 |
| LG1 | Aco012428.1 | | LG9 | Aco017563.1 |
| LG1 | Aco015105.1 | | LG17 | Aco003667.1 |
| LG1 | Aco011341.1 | | LG21 | Aco007999.1 |
| LG2 | Aco001069.1 | | LG6 | Aco003018.1 |
| LG2 | Aco001069.1 | | LG20 | Aco014671.1 |
| LG3 | Aco015492.1 | | LG8 | Aco016643.1 |
| LG4 | Aco022101.1 | | LG13 | Aco013644.1 |
| LG6 | Aco002729.1 | | LG15 | Aco004028.1 |
| LG6 | Aco003018.1 | | LG15 | Aco019842.1 |
| LG7 | Aco004839.1 | | LG9 | Aco017563.1 |
| LG7 | Aco004987.1 | | LG9 | Aco008623.1 |
| LG15 | Aco019842.1 | | LG20 | Aco014671.1 |
| LG22 | Aco017499.1 | | LG24 | Aco013229.1 |
